# The paradigm of drug resistance in cancer: an epigenetic perspective

**DOI:** 10.1042/BSR20211812

**Published:** 2022-04-19

**Authors:** Swagata Adhikari, Apoorva Bhattacharya, Santanu Adhikary, Vipin Singh, Shrikanth S. Gadad, Siddhartha Roy, Chandrima Das

**Affiliations:** 1 Biophysics and Structural Genomics Division, Saha Institute of Nuclear Physics, 1/AF Bidhannagar, Kolkata 700064, India; 2 Homi Bhaba National Institute, Mumbai 400094, India; 3 Structural Biology and Bioinformatics Division, CSIR-Indian Institute of Chemical Biology, 4 Raja S.C. Mullick Road, Kolkata 700032, India; 4 Department of Molecular and Translational Medicine, Center of Emphasis in Cancer, Texas Tech University Health Sciences Center El Paso, El Paso, TX, U.S.A.; 5 Mays Cancer Center, UT Health San Antonio MD Anderson Cancer Center, San Antonio, TX 78229, U.S.A.

**Keywords:** cancer, Cancer stem cells, drug resistance, epigenetics

## Abstract

Innate and acquired resistance towards the conventional therapeutic regimen imposes a significant challenge for the successful management of cancer for decades. In patients with advanced carcinomas, acquisition of drug resistance often leads to tumor recurrence and poor prognosis after the first therapeutic cycle. In this context, cancer stem cells (CSCs) are considered as the prime drivers of therapy resistance in cancer due to their ‘non-targetable’ nature. Drug resistance in cancer is immensely influenced by different properties of CSCs such as epithelial-to-mesenchymal transition (EMT), a profound expression of drug efflux pump genes, detoxification genes, quiescence, and evasion of apoptosis, has been highlighted in this review article. The crucial epigenetic alterations that are intricately associated with regulating different mechanisms of drug resistance, have been discussed thoroughly. Additionally, special attention is drawn towards the epigenetic mechanisms behind the interaction between the cancer cells and their microenvironment which assists in tumor progression and therapy resistance. Finally, we have provided a cumulative overview of the alternative treatment strategies and epigenome-modifying therapies that show the potential of sensitizing the resistant cells towards the conventional treatment strategies. Thus, this review summarizes the epigenetic and molecular background behind therapy resistance, the prime hindrance of present day anti-cancer therapies, and provides an account of the novel complementary epi-drug-based therapeutic strategies to combat drug resistance.

## Drug resistance, a vile opponent to current anti-cancer therapies

In 1971, ‘National Cancer Act’ of the U.S. Congress declared a ‘War on cancer.’ Since then, billions of dollars have been invested in cancer research. Yet, despite decade-long studies and advancement in anti-cancer therapeutic regimens, cancer still poses as one of the major causes of global morbidity and mortality, with the diagnosis of millions of new cases every year. In addition, the development of drug resistance, consequent ineffectiveness of therapy, and successive tumor relapse lead to poor predictive outcomes in the majority of the cases and serve as the major limiting factors of the present day anti-cancer therapies. Studies have shown that despite substantial development in the early detection of breast cancer, more than one-third of patients do not respond to the primary chemotherapeutic regimens and develop therapy resistance [1,2].

Moreover, the fact that chemotherapy is used as the first-line treatment strategy against the majority of the advanced carcinomas only worsens the scenario [3]. Despite the effective reduction in tumor size at the primary site, several patients display the development of distant metastasis [4], which is frequently associated with the development of chemoresistance. It is reported that the selection pressure induced by chemotherapy favors inherently drug-resistant cells (for instance, cancer stem cells (CSCs)) to self-renew, proliferate, and even undergo epithelial-to-mesenchymal transition (EMT) that promotes their metastatic dissemination [5–7]. The tumor cells can also employ intricate signaling cascades to acquire chemoresistant properties during therapy [8,9]. These inherent and acquired chemoresistant cells serve as a repertoire for future tumor recurrence that can give rise to even more aggressive forms of the disease.

The concept of drug resistance was first reported in bacteria, based on antibiotic resistance. Studies on cancer and several other diseases showed similar, evolutionarily conserved mechanisms that impart resistance towards treatment stratagems [10]. For example, therapy resistance in cancer was first noted in the 1940s [11], and over the years, it has become one of the significant concerns of present day cancer management. In addition, a thorough understanding of critical molecular and epigenetic pathways behind this drug-resistant property of cancer cells could unveil a magnitude of possibilities that can be employed to sensitize these resistant cells towards conventional treatment regimens successfully. Finally, a comprehensive overview of the current understanding of the epigenetic mechanisms that render therapy resistance in cancer cells is also elucidated through this review.

## CSCs: the key players behind drug resistance

The recent era of cancer research has shifted a significant focus towards CSCs as they are being reported as one of the major culprits behind maintaining all the hallmarks of cancer. CSCs, a distinct subset of cells within the heterogeneous tumor mass, are adorned with the ability to self-renew and differentiate into multiple lineages [12]. Over the past few years, many studies have established that CSCs are primary therapy-resistant cells within a tumor, which show numerous resistance mechanisms towards radiotherapy and conventional chemotherapeutic regimen [13,14]. The CSCs are usually resistant to chemotherapy/radiotherapy, which drives as a significant cause of cancer recurrence. They survive after the therapeutic intervention, serving as the seed to promote future tumor relapse. Neoadjuvant chemotherapy enriches the CSC population, suggesting a higher degree of resistance to therapy than the rest of the bulk tumor [15]. Interestingly, CSCs are reported to display different mechanisms of therapy resistance, including induction of EMT, high expression of ATP-binding cassette transporters (ABC transporters) and detoxification genes, quiescence, and evasion of apoptosis [16,17].

### EMT

The EMT is the preliminary step in cancer metastasis whose induction is governed by epigenetic modifiers, like in the case of one of the transcription factors, Snail, which recruits multiple chromatin modifiers such as PRC2, histone deacetylase (HDAC)1/2, G9a, LSD1, to the promoter of E-cadherin, thereby promoting Snail-mediated E-cadherin repression, a hallmark of EMT [18].

It has been reported that the induction of EMT promotes the acquisition of stem cell-like features in cancer cells. For instance, Twist, Slug, and Snail, the critical regulators of EMT, induce mesenchymal properties in breast cancer cells, triggering stem cell-like properties and mammosphere-forming ability [19]. It has been suggested that EMT is associated with the induction of drug resistance [7,20]. Previous studies have indicated that the distinct phenotypic differences between CSCs and non-stem cancer cells (NSCCs) are majorly due to the induction of EMT and mesenchymal properties in the CSCs [6]. However, the conventional therapeutic regimens fail to eliminate the cancer cells with CSC-like and mesenchymal properties, promoting CSC-mediated tumor recurrence [7].

Interestingly, EMT and stemness share common signaling pathways like Wnt, Hedgehog, and Notch [20], which suggest their standard mode of action in the induction of drug resistance [21,22]. For example, TGF-β-induced EMT promotes drug resistance [23], while inhibition of TGF-β sensitizes the cells towards chemotherapy [24,25]. Notch 1 signaling-induced EMT activation triggers gefitinib resistance in lung cancer cells [26,27]. The transcription factor ZEB1, which contributes to the regulation of stemness, also plays a significant role in inducing chemoresistance [28]. In pancreatic ductal adenocarcinoma cells, loss of Twist and Snail sensitizes them towards gemcitabine, improving the prognostic outcome [29]. Finally, mechanistically stemness, EMT, and drug resistance are linked, and understanding this network is crucial for developing more potent therapeutic strategies.

### Increased expression of ABC transporter and detoxification genes

ABC transporters like ABCB1 (MDR1), ABCC1 (MRP1), ABCG2 are transmembrane proteins that pump toxins out of the cells using energy from ATP hydrolysis. CSCs of various solid tumors display significantly high expression of these drug efflux pumps, rendering them intrinsically resistant to most conventional therapeutic interventions [30,31]. ABCG2 has been reported to efflux the chemotherapeutic drugs doxorubicin and methotrexate [32] and its down-regulation enhances the chemosensitivity of breast CSCs [33]. Importantly, in glioma tumor stem-like cells, PI3K/Akt signaling promotes localization of ABCG2 to the plasma membrane [34]. Moreover, CSC marker aldehyde dehydrogenase 1 (ALDH1), which catalyzes the oxidation of aldehydes, protects them from reactive oxygen species (ROS)-mediated damages [35]. It is also reported that lung carcinoma stem cells showing elevated expression of ALDH1 confer resistance towards gefitinib, an epidermal growth factor receptor tyrosine kinase inhibitor (EGFR-TKI) and other chemotherapeutic drugs [36].

### Dormancy or quiescence

Chemotherapy and radiation therapy effectively damages the proliferating tumor cells. However, they have little effect on the cells in dormant/quiescent state [37]. The quiescent state is a state of reversible cell cycle arrest, where the cells remain in the G_0_ phase and require a mitogen stimulus to enter into the division phase [38]. In the core tumor, CSCs often remain in the state of dormancy, which helps them avoid being targeted by drugs and may give them time for efficiently repairing the damaged DNA. Interestingly, in a study in the human bladder cancer xenograft model, between the gap of chemotherapy cycles, quiescent-label retaining CSCs got recruited into cell division in response to the drug-induced damage. This shows similarity to the recruitment of normal stem cells during the wound-healing process and indicates the role of CSCs in the development of therapy resistance [39].

### Evasion of apoptosis

The ability of CSCs to evade apoptosis, a major hallmark of cancer, is well established. Different signaling pathways such as Hedgehog and Notch signaling are activated in CSCs that reportedly promote anti-apoptosis, linked to docetaxel resistance in prostate cancer [40]. Additionally, CSCs can survive DNA damage-mediated apoptosis in various ways. For example, the guardian of genome p53 gets activated in response to DNA damage, and depending on the extent of damage, it positively regulates apoptosis. But in CSCs, p53 is either down-regulated or mutated, which is positively associated with survival of CSCs through dysregulation of apoptosis [41]. Besides, CSCs have a well-developed free radical scavenging system similar to normal stem cells to minimize the level of intracellular ROS, which are considered mediators of ionizing radiation-driven cell death, leading to resistance towards radiotherapy [42].

## The epigenetic scenario behind different drug resistance mechanisms in cancer

Several studies have established the significance of epigenetic changes in cancer drug-tolerant persister (DTP) cells which survive within the heterogeneous population and evolve to be more tolerant to increased drug pressure [43–45]. Furthermore, it has been indicated that modulation of epigenetic landscape contributes to the survival and maintenance of CSCs [46]. In addition, cancer cells reorient their epigenomic landscape through DNA methylation and modifications of histone and non-histone proteins to develop different drug resistance mechanisms to avert anti-cancer therapies. Other epigenetic regulators such as DNA methyltransferases (DNMTs), chromatin readers, writers and erasers, various histone modifiers and non-coding RNAs fine-tune the regulation of genes involved in multiple modes of therapy resistance.

### Elevated expression of drug efflux pumps, the role of epigenetics

High efflux of therapeutic agents leading to reduced intracellular drug accumulation is established to be the critical reason behind therapy resistance. The ability to efflux drugs at a significantly high rate, to render resistance may be either intrinsic to the tumor or acquired following an initial cycle of therapy. The ABC transporter superfamily, which includes ABCG2, MRP1, and MDR1, are the primary transmembrane transporters responsible for drug efflux [47]. These drug efflux pumps can pump out various therapeutic drugs such as anthracyclines, alkylating agents, vinca alkaloids etc.

**Figure 1:**
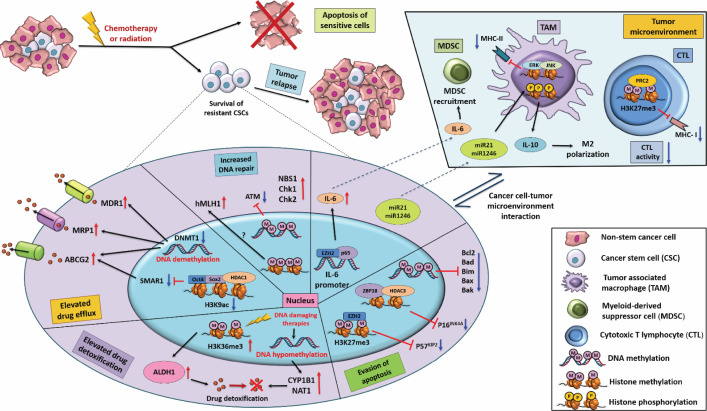
The epigenetic regulations behind the different mechanisms of drug resistance Schematic diagram representing the different epigenetic alterations that render increased drug efflux, elevated drug detoxification, augmented DNA repair, and apoptosis resistance properties to the cancer cells, making them resistant towards conventional therapeutic regimens. The interaction between tumor microenvironment and cancer cells, which shapes tumor survival and progression, is also represented schematically.

CSCs are reported to display the highest expression of these drug efflux pumps within the tumor mass, which is attributable to their resistant phenotype [48]. A recent study has reported that Oct4 and Sox2, the two major stemness factors, indirectly up-regulate ABCG2 expression in CSCs by repressing SMAR1 via recruitment of HDAC1 which leads to deacetylation of SMAR1 promoter (Figure 1). In the absence of Oct4 and Sox2, SMAR1 represses ABCG2 by directly recruiting HDAC2 on its promoter [49]. Also, the high expression of ABC transporters in CSCs is associated with promoter hypomethylation [50]. Thus, promoting DNMT activity in CSCs might be a potential tool to chemosensitize them. For example, a study by Wang et al. has demonstrated that increasing DNMT activity by afatinib results in hypermethylation of the ABCG2 promoter and lowers ABCG2 expression in drug-resistant breast cancer cells [51]. Another study by Martin et al. reported that inducing DNMT activity in brain CSCs increases ABCG2 promoter methylation and represses its expression [52].

Moreover, the increased expression of ABCG2 and the reduced folate carrier SLC19A1 is reported to be due to promoter hypomethylation, which positively correlates with induction of drug resistance [53,54]. Furthermore, the expression of Phase-III transporters such as ABCC6 and SLC22A3 is also modulated by alterations in DNA methylations [55]. Interestingly, in triple-negative breast cancer, the chromatin reader and tumor suppressor protein ZMYND8 promote the formation of a transcription repressor complex with KDM5C and EZH2 that increases the H3K27me3 mark on promoters of ABCB1, ABCC1, and ABCC2 drug efflux pumps, thereby repressing their expression [56]. These pieces of evidence indicate the ability of CSCs to promote drug resistance by epigenetic modifications of DNA and chromatin landscape for up-regulating drug efflux pump expression.

### Epigenetic regulation in drug detoxification

Drug-metabolizing enzymes (DMEs), including phase-I and phase-II DMEs, are responsible for biotransformation and detoxification. In phase-I reaction, the drugs are hydrolyzed and detoxified by DMEs like cytochrome P450 enzymes, aldehyde dehydrogenases etc. [57]. While phase-II DMEs catalyze the conjugation of the hydrophilic compound with the products of phase-I reaction making the latter water-soluble compound for easy excretion. Phase-II DMEs consist of transferases such as glutathione S-transferase (GST) [57]. Epigenetic interplay differentially regulates the DME genes in cancer, mediating their drug resistance potential. In addition, CSCs display a significant increase in drug detoxification machineries, promoting their ‘non-targetable’ nature. CSCs are associated with high expression of detoxifying enzymes like ALDH1, which is used to identify CSCs [58]. A report by Honoki et al. demonstrated that the CSCs residing in tumorospheres possess high ALDH1 activity and show the robust capability of chemoresistance and drug detoxification [59] (Figure 1). Another recent study by Peitzsch et al. demonstrated that chemotherapy and irradiation lead to an enrichment of H3K36me3 transcription activation mark on ALDH1 promoter, which up-regulates ALDH1 expression and promotes resistant CSC-like phenotype in the treated cells [60].

P450 is an abundant DME family, subdivided into two subfamilies CYP1-4 and CYP7-51 [57]. Genome-wide integrative analysis established that the DME genes such as *CYP2D6, SULT1A1, GSTM5, CYP2C19, CYP1A2, GSTT1*, and *GSTA4* are differentially modulated by DNA methylation in cancer which leads to inter- and intra-individual differences in drug metabolism, ultimately correlating to poor prognostic outcome [61]. Interestingly, in prostate cancer, CYP24A1, another cytochrome P450 family enzyme, is repressed by promoter methylation and histone repressor mark H3K9me2 [62]. The methylation landscape of the promoters of DMEs predicts prognostic outcome and treatment efficacy of hormone-responsive breast cancers. For instance, the promoter methylation status of estradiol and tamoxifen-metabolizing enzyme CYP1B1 from the P450 family has been reported to predict survival and prognosis in tamoxifen-treated and untreated patients [63]. Another example in this context is the hypermethylation of a phase-II DME, N-acetyl transferase 1 (*NAT1*) gene, which regulates tamoxifen resistance in breast cancer [64].

### The epigenetic mechanisms behind heightened DNA repair

The DNA repair pathway responds to DNA damage and maintains genomic integrity. The DNA repair machinery comprises an intricate system of sensors, transducers, and effectors that synchronize the repair of damaged DNA, thus ensuring cellular survival [65]. The compaction of chromatin regulates the accessibility of DNA repair and other cellular machineries [66,67]. So, by modulating the chromatin compaction, cancer cells modulate the impact of damaging agents over the exposed DNA and subsequently regulate the DNA repair mechanism [68]. It is indicated that the level of radiation-induced double-strand break (DSB) was 5–50 folds higher in the euchromatin region as compared with the heterochromatin region, and also the euchromatin is more prone towards the regional mutation rate variation [69,70]. Accumulating pieces of evidence of the past few years have indicated cancer cells display altered DNA repair pathways that play a crucial role in their resistance towards genotoxic drugs [71]. A study by Kandoth et al. using next-generation sequencing-based large-scale mutation mapping in cancer has unravelled that cancer cells show an elevated rate of gene mutations that play a role in DNA and histone alterations and chromatin remodeling [72], thus leading to possible epigenomic alteration in cancer cells. This altered epigenetic landscape in cancer cells leads to global changes in histone modification patterns, DNA methylation, and nucleosome positions, affecting their chromatin architecture [73]. These, in turn, alter the DNA damage response and repair in cancer cells, which can either allow accumulation of heightened DNA damage, causing instability in the genome (a hallmark of cancer), or can promote enhanced DNA repair, rendering resistance to genotoxic drugs.

The DNA repair protein Ataxia Telangiectasia Mutation (ATM) functions to sense DNA damage and simultaneously activate the repair mechanism associated with it, including homologous recombination and non-homologous end-joining methods [74]. In addition, ATM is a critical player in DNA DSB repair induced by chemotherapeutic treatment, ionizing radiation etc. In addition, ATM gene aberration and epigenetic alteration of ATM expression in cancer are routinely associated with chemotherapy resistance and poor prognostic outcome [75]. Hypermethylation in the promoter of hMLH1, a protein involved in mismatch repair, is commonly seen in colorectal cancer [76] (Figure 1). 5-Aza-2′-deoxycytidine (decitabine)-mediated demethylation of hMLH1 gene promoter leads to sensitization of colorectal cancer cells towards chemotherapeutic drug, 5-fluorouracil [77].

The CSCs show an even more improved rate of DNA repair, which allows them to be the most drug-resistant cells within the heterogeneous tumor mass [78]. The high expressions of O(6)-methylguanine-DNMT, NBS1, Chk1, and Chk2 in CSCs in comparison to NSCCs renders them more resistant towards DNA-damaging therapies, and the altered expression of these genes in CSCs can be attributed to their differential epigenetic landscape [37,79].

### The epigenetic allies in inhibiting apoptosis

Tumor development, progression, and response to therapeutic interventions are tightly regulated by programmed cell death or apoptosis [80]. Evading apoptosis is one of the main hallmarks of cancer, ensuring cancer cell survival upon exposure to cytotoxic and genotoxic stresses [81]. Since the mechanism of action of most of the current treatment regimens like chemotherapy is to activate apoptotic pathways, resistance towards apoptosis shapes therapeutic resistance to a massive extent [82]. The acquisition of apoptosis resistance depends on various factors, including equilibrium of pro- and anti-apoptotic signals and expression of the apoptosis-related genes [83]. The different pro- and anti-apoptotic signaling factors are regulated epigenetically at transcription and post-translation levels in the cancer cells. For instance, enhanced expression or activity of anti-apoptotic factors like Bcl-xl, Bcl-2, IAPs etc. leads to the development of apoptosis resistance. Similarly, repression of pro-apoptotic factors like Bax, Bid, Puma, Noxa, Bim etc. promotes resistance towards apoptosis. Moreover, Akt and their transcriptional regulators, NF-κB and STAT, remain highly overexpressed in various advanced cancers, indicating their possible involvement in therapy resistance [84].

Different histone variants and their modifications are associated with carcinogenesis and therapy resistance. For example, the histone variant H2AZ is reported to be overexpressed in various solid cancers, contributing to tumor progression [85]. Knockdown of H2AZ leads to repression of Bcl-2 and elevation of pro-apoptotic marks Caspase 3, Caspase 9, and Bak, thus stimulating apoptotic cell death [86]. Aberrant histone alterations are often associated with cancer progression and therapy resistance. Previous studies have indicated the involvement of dephosphorylation of histone H1, phosphorylation of H2A, H2B, H3, and H4, and deubiquitylation of H2A in the cascade of apoptosis [87]. Tumor cells resist drug toxicity-mediated apoptosis by up-regulating the expressions of various DNA repair genes (like *MGMT, BRCA 1/2* etc.) through activation of H3K4Me3 [83]. A study in human lung cancer cell line NCI-H460 revealed that repression of *p16^INK4a^* promoter by cumulative action of ZBP-89 and HDAC3 regulates senescence and apoptosis [88] (Figure 1). In breast carcinoma, *CDKN1C* (the gene encoding tumor suppressor p57^KIP2^) is repressed by EZH2-mediated histone H3K27 trimethylation (H3K27me3), leading to the acquisition of a more resistant phenotype [89] (Figure 1). In bladder carcinoma cells, the repression of the *p21^WAF1^* promoter is mediated by histone deacetylation through the activity of HDACs [90]. This leads to the development of apoptotic resistance, which can be effectively reversed by targeting the cells with HDAC inhibitors (HDACis) [90]. In addition, specific histone modifications can lead to the recruitment of protein complexes governing the balance between cell death and survival. For example, phosphorylation of H2A.X-Y142 represses MDC1-induced recruitment of DNA repair proteins to DNA damage site (yH2AX) [91]. This, in turn, promotes the recruitment of pro-apoptotic complexes to the site, pushing the cell towards apoptotic fate [91,92]. During the acquisition of therapy resistance, this pathway alters in the cancer cells, thus resisting cell death even after being treated with DNA damaging agents like chemotherapy.

DNA methylation status also plays a pivotal role in modulating apoptotic cell fate in various cancers. The CpG islands on the promoters of tumor suppressor and pro-apoptotic genes remain hypermethylated by the activity of DNMT in cancer cells [93] (Figure 1). Hypermethylation of these genes inhibits the programmed cell death pathway, rendering the tumor cells a resistant phenotype. For instance, the promoter of pro-apoptotic markers Bcl-2 and Bik reportedly remain hypermethylated in prostate cancer [94,95]. Similarly, in multiple myeloma, the promoters of *Bad, Bax, Bak*, and *Puma* display hypermethylation [96]. The promoter of *Bim* reportedly shows hypermethylation and repression in chronic myeloid leukemia [97]. Promoter methylation and repression of Caspase 8 and 10, two significant contributors of the apoptotic pathway, render resistant phenotype in bladder cancer, hepatocellular carcinoma, glioblastoma, small-cell lung cancer, retinoblastoma, and neuroblastoma [98–102]. In gastric cancer, the expression of *Bcl-2l10, BNIP3*, and *HRK* remains inhibited by promoter hypermethylation [103–105]. p53, the master tumor suppressor, plays a pivotal role in initiating the apoptotic cascade. In patients with acute lymphatic leukemia, the p53 gene remains hypermethylated and inhibited, leading to the ineffectiveness of treatment regimens [106]. Also, repression of hypermethylated in cancer 1 (HIC1) in cancer cells leads to inactivation of p53, thus inhibiting DNA damage-mediated apoptosis pathway [107]. MGMT, MLH1, BRCA1, APC, and APAF1 expressions are also reported to be diminished in cancer cells by hypermethylation of their promoters [108,109]. Repression of *FAS* expression by hypermethylation in its promoter is reported to be involved in cutaneous T-cell lymphoma carcinogenesis and apoptosis resistance [110].

Apart from DNA methylation and histone post-translational modifications, miRNAs also play a critical role in the modulation of apoptosis resistance. For instance, p53 induces the expression of the miR-34 family, which reportedly can inhibit the expression of MYCN, CDK4/6, Notch, Cyclin E2, and Bcl-2 [111]. The expression of miR-34 is repressed in various solid and liquid cancers, including breast/colon/gastric/kidney/pancreatic carcinoma, Burkitt’s lymphoma, and chronic lymphocytic leukemia indicating the ingenious strategy of cancer cells to resist apoptosis [112,113]. Similarly, the expressions of miR-15, miR-16, miR-193a-3p, miR-29b, miR-133b, miR-512-5p remain repressed in different types of cancers due to their involvement in promoting apoptosis [114–119]. In contrast, miR-221 and miR-222 remains overexpressed in various solid cancers, leading to repression of TIMP3, PTEN, FOXO3A, PUMA, Caspase 3, etc. [120–123]. miR-BART5 is reported to be involved in the apoptosis-resistance of gastric cancer cells due to its ability to target PUMA [124]. miR-135a, which reportedly represses JAK2 and Bcl-xl, displays down-regulation in various cancers like ovarian cancer, AML etc. [125,126].

All these reports indicate the intricate involvement of epigenetic machinery in modulating apoptosis in cancer cells, indicating that epigenetic reprogramming plays a pivotal role in rendering apoptosis resistance to the cancer cells.

## The role of tumor microenvironment in supporting tumor progression and mediating drug resistance

The tumor microenvironment (TME) is an intricate and dynamic landscape that consists of proliferating tumor cells, tumor-associated immune cells, endothelial cells, and extracellular matrix. The TME plays a crucial part in tumor progression, invasiveness, metastatic insemination and impacts the clinical outcome of the tumor. The interaction between the cell and extracellular matrix of the TME is critical for cancer progression, and it plays a vital role in the development of therapy resistance. The different components of TME are reported to be regulated and reprogrammed by epigenetic alterations, including DNA and histone modifications.

### Role of epigenetics in reprogramming tumor-associated immune landscape

It is well established that epigenetic machinery plays a critical role in reprogramming and recruiting the intrinsic and adaptive immune cells into the TME, supporting tumor progression. It is reported that HDAC5-mediated inhibition of Socs3 and up-regulation of CCL2 leads to recruitment of tumor-associated macrophages (TAMs) [127]. In the TAMs, ERK and JNK facilitated histone deacetylation of CIITA promoter leads to decoy receptor (DcR3)-induced repression of MHC-II expression [128]. This impairs antigen presentation in TAMs, resulting in immunosuppression in the TME [128]. In addition, the myeloid-derived suppressor cells (MDSCs) are a subset of immune cells that expands during cancer and plays a critical role in tumor development, progression, and therapy resistance [129]. It is reported that, in hepatocellular carcinoma, the interaction between EZH2 and p65-NF-kB and their binding on IL-6 promoter augments its expression and induces MDSC recruitment in the TME, which is correlated with a poor therapeutic response [130] (Figure 1).

Furthermore, the CD8+ cytotoxic T cells recognize and kill target antigens through MHC-I, which is epigenetically repressed in the TME as a strategy for immune evasion. Finally, a study has reported that PRC2 suppresses MHC-I through bivalent H3K4me3 and H3K27me3 modifications [131] (Figure 1). Moreover, the expression of DNMT1 and EZH2 shows a negative correlation with CD8+ T-cell infiltration within the TME and consequently the patient’s clinical outcome [132].

MicroRNAs also play a distinct role in shaping the immunosuppressed environment within the TMEs, thereby promoting tumor progression. For instance, the repression of miR-29 in cancer promotes augmentation of B7-H3, resulting in dysfunction of Natural Killer (NK) cells and immune evasion [133]. Also, the inhibition of perforin and granzyme expression by miR-27a* reduces anti-cancer cytotoxic functions of NK cells and cytotoxic T cells within the TME [134].

### The role of TME components in tumor progression

A study by Hanson et al. in prostate cancer revealed differential promoter methylation status of various genes in epithelial and stromal cells in benign and malignant tumors [135]. It was shown that a distinct promoter methylation pattern in stromal cells is correlated with tumor development. The TME also plays a critical role in inducing intrinsic resistance to chemotherapy via ‘reversed pH gradient,’ pumping out protons through proton transporters compared with normal cells, which have low intracellular pH compared with extracellular pH [136]. The TME also adapts to dynamic changes as therapy continues, which leads to a gain in resistance to both chemo and targeted drugs. For instance, the CSC component of the TME plays a critical role in developing therapy resistance. The CSCs survive after radio or chemotherapy and shows resistance even towards one of the most novel and selective therapeutic system, immunotherapy [137]. The NSCCs, CSCs, and immune components of the TME, through their secretions, reciprocate feedback to each other to overcome therapy-induced stress and increase survival [138].

Intricate epigenetic regulatory pathways within the different components of TME play a crucial role in ensuring tumor progression. For example, in TAMs, activating epigenetic marks like histone phosphorylation by ERK-1/2 at the IL-10 promoter results in elevated production of IL-10, which stimulates immunosuppression [139]. Moreover, in TAMs, Tet2 reportedly sustains its immunosuppressive and tumor-promoting activity through DNA methylation [140]. In triple-negative breast cancer, LSD1 demethylase is allegedly involved in M2 polarization by activating the expression of IL-1b, IL-12b, IL-8, NOS2, CCR7, Gpr18 etc. [141]. Also, exosomal miR-21 and miR-1246 can enhance the production of IL-10 and TGF-β in primary human macrophages, leading to M2 activation [142] (Figure 1). M2 macrophages are established to have pro-tumor functions and have previously been associated with the acquisition of drug resistance [143]. In chemoresistant acute lymphoblastic leukemia, the interaction of cells within the bone marrow microenvironment is reported to be facilitated by epigenetic modifiers. Inhibition of these interactions using DNMT inhibitor azacitidine and HDACi panobinostat reportedly chemosensitizes these cells [144].

The components of core TME undergo hypoxic stress that causes elevated expression of hypoxia-inducible factor 1 α (HIF-1α). In hypoxic TME, reduced hydroxylation of HIF-1α favors its stabilization. Also, post-translational modifications of HIF-1α, including phosphorylation and SUMOylation have been reported to promote its stabilization [145,146]. Overexpression of HIF-1α orchestrates the transcription of various genes leading to the alteration in metabolism, activation of angiogenesis and metastasis, and the development of chemoresistance [147–149]. In addition, several microRNAs are reported to play an intricate part in the hypoxia-mediated acquisition of drug resistance. For instance, down-regulation of miR-15 and miR-16 due to hypoxia is associated with chemoresistance [150,151]. HIF-1α transcriptionally activates the expression of miR-20a, an elevated level of which has been found to induce therapy resistance [152,153]. Also, hypoxia-regulated expression of miR-200b reportedly promotes chemoresistance in cholangiocarcinoma [154].

All these studies cumulatively point towards the intricate involvement of epigenetic machinery in reprogramming and utilizing TME in a pro-tumor manner.

## Alternative treatment strategies to sensitize the drug-resistant cells

For advanced stages of cancer, a combination of chemotherapeutic agents is often favored over single agents to achieve a higher and quicker therapeutic response. Combination chemotherapy regimens are reported to show a better response in the case of tumor regression [155]. For instance, FAC (5-fluorouracil+adrenamycin+cyclophosphamide) and FEC (5-fluorouracil+adrenamycin+cyclophosphamide) are routinely used combinations for breast cancer. In metastatic breast carcinomas, Gemcitabine has also been utilized with paclitaxel for clinical trials [156]. CHOP (cyclophosphamide, doxorubicin hydrochloride, vincristine, and prednisone) is one of the most routinely used therapy in diffuse-large B-cell lymphoma [157]. However, combining two or more chemotherapeutic agents increases the amount of systemic toxicity and increases the chance of chemotherapy-related complications. To address this issue, several studies have utilized other drugs, small molecules, or pathway inhibitors in complement with chemotherapy to sensitize the drug-resistant cells. In recurrent ovarian cancers, VEGF and PARP inhibitors are combined with chemotherapeutic drugs [158]. The anti-diabetic drugs thiazolidinedione and metformin have been repurposed to target the chemoresistant CSCs by several studies, in which these drugs significantly sensitized the CSCs towards chemotherapy [159,160]. Curcumin, an active component obtained from turmeric (*Curcuma longa*) rhizome, has been well established by several studies as a potent chemosensitizing agent for various cancers [161–163]. The role of NFκB in the acquisition of drug resistance is well recognized by several studies [164,165]. The commonly used NSAID aspirin, due to its ability to inhibit nuclear transport of NFκB, has been extensively utilized by several studies to target cancer cells and CSCs [49,166,167]. Aspirin pre-treatment has been shown to suppress the acquisition of chemoresistance in breast CSCs [168]. Quite a handful of studies have pointed to the efficacy of using various miRNAs combined with chemotherapy to sensitize chemoresistance in cancer [169–172]. However, cancer cells’ extensive epigenetic reprogramming ability poses a major challenge to all the combinatorial approaches and even increases the possibility of acquiring resistance towards these therapies.

## Combating drug resistance by targeting epigenetic modifiers

**Figure 2:**
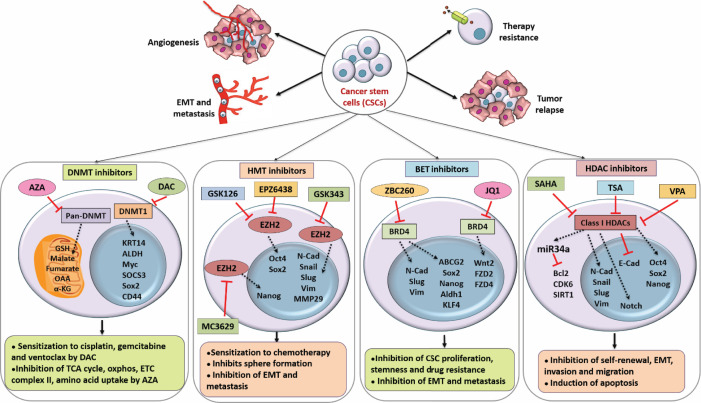
Epidrugs are used to target and sensitize CSCs Several studies have utilized different epigenetic modulators including DNMT inhibitors, HMT inhibitors, BET inhibitors, and HDACis, to target therapy resistance, self-renewal, proliferation, and migratory potential of CSCs and to sensitize them towards chemotherapy. Abbreviation: AZA, azacytidine; DAC, decitabine; E-Cad, E-cadherin; HMT, histone methyltransferase; N-Cad, N-cadherin; OAA, oxaloacetic acid; SAHA, suberoylanilide hydroxamic acid; TSA, trichostatin A; Vim, vimentin; VPA, valproic acid; α-KG, α-ketoglutarate.

Various studies so far have established that in addition to genetic mutations, epigenetic modifications play a pivotal role in acquiring chemoresistance in cancer cells. However, the epigenetic alternations are reversible and require to be actively maintained by epigenetic modifiers, making them an attractive target for therapeutic intervention. The current understanding of CSC epigenome provides new insights into targeted therapy by epigenetic drugs to overcome CSC drug resistance [173]. Several studies have utilized epigenome modifying drugs alone or combined with conventional treatments to modulate the epigenetic changes to attenuate drug resistance [174] (Figure 2). Although these epigenome-altering drugs are ‘non-specific’ in nature and may affect global gene expression, it is also reported that they can cause local changes in gene expression depending on the gene chromatin environment [175]. Furthermore, it is reported that the sensitivity towards epigenetic alternators can be genomic loci-specific depending on the three-dimensional structure of chromatin [175].

DNMTs, HDACs, and histone methyltransferases (HMTs) are among the most widely targeted epigenetic modifiers in contemporary anti-cancer therapies. Inhibitors of DNMTs, HDACs, histone demethylases (HDMs), HMTs and bromodomain proteins are used individually or in combinations to various clinical trials with or without chemotherapy [176–178]. Table 1 enlists different types of epigenome-modifying drugs and their utilization, targeting multiple types of solid cancers and hematological malignancies.

**Table 1:** Various epigenetic modulators in anti-cancer studies and clinical trials

Type	Compound	Activity	Studies done in cancers	Status of clinical trial
DNMT inhibitors	Azacitidine	Inhibits DNMT activity	AML [179], prostate [180], multiple myeloma [181], non-small cell lung carcinoma (NSCLC) [182]	• AML: Phase III (completed- NCT00887068; active- NCT01757535) • Prostate cancer: Phase II (Completed- NCT00384839) • MDS: Phase III and IV (completed- NCT00071799, NCT01201811) • Pancreatic cancer: Phase II (Recruiting- NCT01845805) • CML: Phase II (completed-NCT01350947) • Refractory T-cell lymphoma: Phase III (recruiting- NCT03703375) • Head and neck squamous cell carcinoma: Phase II (recruiting- NCT02178072)
	Decitabine	Inhibits DNMT1 activity	Bladder carcinoma [183], glioblastoma [184,185], hepatocellular carcinoma [186], renal cell carcinoma [187], acute leukemia [188]	• AML: Phase II (completed- NCT00416598) • Refractory CML: Phase II (completed- NCT00042003) • MDS: Phase II and III (completed- NCT00067808 and NCT00043381) • Refractory diffuse-large B-cell lymphoma: Phase IV (recruiting- NCT03579082) • Refractory T lymphoblastic lymphoma: Phase IV (recruiting- NCT03558412) • Follicular thyroid cancer: Phase II (completed- NCT00085293)
	Procaine	Prevents DNMT1 and 3A from binding to DNA	Gastric cancer [189], nasopharyngeal cancer [190]	• Nasopharyngeal neoplasms: Phase II (mixture of Procaine with dexamethasone, gentamicin and vitamin B12; unknown status-NCT02735317)
	Zebularine	Inhibits DNMT activity	Ovarian cancer [191]	No clinical trial yet
HDACi	Vorinostat/SAHA	Inhibits class I and II HDACs	Leukemia [192], glioblastoma multiforme (GBM) [193], breast cancer [194,195], melanoma [196], pancreatic cancer [197], neuroblastoma [198], retinoblastoma [199], NSCLC [200]	• GBM: Phase II (vorinostat + temozolomide + radiation, active- NCT00731731) • Advanced thyroid cancer: Phase II (completed- NCT00134043) • Kidney cancer: Phase II (completed- NCT00278395) • Advanced NSCLC: Phase II (completed- NCT00138203) • Progressive GBM: Phase II (completed- NCT00238303) • Breast cancer: Phase II (vorinostat + tamoxifen, completed- NCT00365599 • Progressive metastatic prostate cancer: Phase II (completed- NCT00330161) • Advanced malignant pleural mesothelioma: Phase III (completed- NCT00128102)
	Abexinostat	Inhibits class I and II HDACs	Nasopharyngeal carcinoma [201], breast cancer [202]	• Renal cell carcinoma: Phase III (active, recruiting- NCT03592472) • Refractory follicular lymphoma: Phase II (active, not recruiting- NCT03600441) • Non-Hodgkin’s lymphoma: Phase II (active, recruiting- NCT04024696)
	Belinostat	Pan HDACi	NSCLC [203], Non-Hodgkin’s lymphoma [204], AML [205]	• Refractory peripheral T-cell lymphoma: Phase II (completed- NCT00865969) • Liver cancer: Phase I/II (completed- NCT00321594) • MDS- Phase II (completed NCT00357162) • Advanced multiple myeloma: Phase II (completed-NCT00131261)
	Panobinostat	Pan HDACi inhibitor	NSCLC [206], [207], diffuse large B-cell lymphoma [208], AML and MDS [209–211], ovarian cancer [212]	• High-risk MDS, AML: Phase I/II (active, not recruiting-NCT01451268) • Metastatic thyroid cancer: Phase II (completed- NCT01013597) • Refractory prostate cancer: Phase II (completed- NCT00667862) • Refractory colorectal cancer: Phase II (completed-NCT00690677) • Refractory CML: Phase II/III (completed- NCT00449761) • HER2-negative locally recurrent or metastatic breast cancer: Phase II (completed- NCT00777049)
	Valproic Acid	Pan inhibitor, binding to the catalytic center of the HDACs	Melanoma [213,214], colon cancer [215], Prostate cancer [216], Breast cancer [217,218], lung cancer [219], head and neck cancer [220], thyroid cancer [221,222]	• Melanoma: Phase II (active, recruiting- NCT0206858) • Colorectal cancer: Phase I/II (valproic acid + radiation therapy, recruiting- NCT01898104) • Advanced thyroid cancers: Phase II (completed- NCT01182285) • High-grade gliomas, brain tumors: Phase II (Valproic acid + temozolomide + radiation, completed- NCT00302159) • NSCLC- Phase I/II (valproic acid + chemoradiotherapy, status unknown- NCT01203735 • Pancreatic cancer: Phase II (valproic acid+ chemotherapy, status unknown- NCT01333631)
	Phenylbutyrate	Pan HDACi	Renal cell carcinoma [223], NSCLC [224]	• Progressive or recurrent brain tumors: Phase II (completed-NCT00006450)
	Entinostat	Class 1 HDACi	Breast cancer [225,226], Renal cell carcinoma and prostate cancer [227]	• Advanced breast cancer: Phase III (recruiting- NCT03538171), Phase II (completed- NCT00676663) • MDS, AML, ALL: Phase II (completed- NCT00462605)\ • Metastatic melanoma: Phase II (completed- NCT00185302)
	Givinostat	Class 1 and 2 HDACi	NSCLC [228]	• Chronic myeloproliferative neoplasms: Phase II (active, not recruiting- NCT01761968)
	Romidepsin (FK228, Depsipeptide)	Class 1 HDACi	Clear cell renal cell carcinoma (ccRCC) and triple-negative breast cancer (TNBC)[229], lung cancer [230], breast cancer[231], bladder cancer[232], T-cell lymphoma [233]	• Progressive peripheral T-cell lymphoma: Phase II (active, not recruiting- NCT00426764, completed NCT00007345) • Metastatic breast cancer: Phase II (completed- NCT00098397) • Relapsed small-cell lung cancer: Phase II (completed-NCT00086827) • Recurrent high-grade gliomas: Phase I/II (completed- NCT00085540) • Relapsed or refractory multiple myeloma: Phase II (completed-NCT00066638)
	Trichostatin A (TSA)	Class 1/3/4/6/10 HDACs inhibitor	NSCLC[234], prostate cancer [235], bladder cancer [236], CML[237], glioblastoma [238]	• Relapsed or refractory hematologic malignancies: Phase I (recruiting-NCT03838926)
HDM inhibitors	Pargyline	Inhibits lysine-specific demethylase 1 (LSD1)	Breast cancer [239], prostate cancer [240]	No clinical trial yet
	HCI-2509	Inhibits LSD1	Lung adenocarcinoma [241]	No clinical trial yet
	S2101	Inhibits LSD1	Ovarian cancer [242]	No clinical trial yet
	MC3324	Inhibits LSD1 and LSD6A	Breast cancer [243]	No clinical trial yet
	Methylstat	Inhibits LSD4B	Breast cancer [244]	No clinical trial yet
	JIB-04	Pan inhibitor of Jumanji-domain histone demethylases	Ewing sarcoma [245]	No clinical trial yet
HMT inhibitors	Tazemetostat	Inhibits EZH2	Solid tumors and hematological malignancies [246], refractory follicular lymphoma [247, 248], thyroid cancer [249], colorectal cancer [250], breast cancer [251]	• Malignant mesothelioma: Phase II (completed- NCT02860286) • Metastatic prostate cancer: Phase I (recruiting, NCT04179864, NCT04846478) • Solid tumors harboring an ARID1A mutation: Phase II (not yet recruiting- NCT05023655) • Refractory INI1-Negative tumors or synovial sarcoma: Phase I (completed- NCT02601937) • Peripheral nerve sheath tumor: Phase II (recruiting- NCT04917042) • Metastatic melanoma: Phase II (recruiting- NCT04557956) • Advanced colorectal carcinoma, advanced soft-tissue sarcoma, advanced pancreatic adenocarcinoma: Phase II (recruiting- NCT04705818)
	CPI-1205	Inhibits EZH2	Small intestinal neuroendocrine tumors [252], B-cell lymphomas [253]	• B-cell lymphoma: Phase I (completed- NCT02395601) • Metastatic castration-resistant prostate cancer: Phase I/II (active, not recruiting- NCT03480646)
	PF-0682149	Inhibits EZH2	Brain cancers [254]	• Small-cell lung cancer, follicular lymphoma and castration-resistant prostate cancer: Phase I (active, recruiting- NCT03460977)
	EPZ-5676 (pinometostat)	Inhibits DOT1L	Leukemia [255,256]	• Refractory leukemias bearing a rearrangement of the MLL gene: Phase I (completed- NCT02141828)
BET inhibitors	JQ1	Inhibits BET proteins BRD2, BRD3, BRD4, and BRDT	Nuclear protein in testis (NUT)-midline carcinoma (NMC) [257], AML [258], medulloblastoma [259], breast cancer [260], and lung cancer [261]	• No clinical trial due to low oral bioavailability
	JQ1 analog RO6870810 (TEN-010/JQ2)	Inhibits BET proteins BRD2, BRD3, BRD4, and BRDT	Advance multiple myeloma [262], AML and MDS [263]	• AML and MDS: Phase I (completed- NCT02308761) • NUT carcinoma, diffuse large B cell lymphoma and other advanced solid tumors: Phase I (completed- NCT01987362)
	OTX015	Inhibits BET proteins BRD2, BRD3, BRD4	Neuroblastoma [264], mesothelioma [265], multiple myeloma [266], and B-cell lymphoma [267]	• AML, diffuse large B-cell lymphoma, ALL, multiple myeloma: Phase I (completed- NCT01713582)
	I-BET62 (Molibresib, GSK525762)	Inhibits BET proteins BRD2, BRD3, BRD4	Multiple myeloma[268], pancreatic adenocarcinoma [269], and neuroblastoma [270]	• Metastatic breast cancer: Phase I (completed- NCT02964507) • Castrate-resistant prostate cancer: Phase I (completed- NCT03150056) • Refractory hematologic malignancies: Phase II (completed- NCT01943851)

### Use of DNMT inhibitors in hindering chemoresistance

DNMT inhibitors are extensively used as tools for hypomethylating the genome in preclinical and clinical studies and the treatment of different types of cancers. Currently, two DNMT inhibitors 5-Azacytidine (AZA, vidaza) and its deoxyribose analog, 5-aza-2′-deoxycytidine (decitabine), are approved by Food and Drug Administration (FDA) as anti-cancer agents. AZA and decitabine are the only epigenetic modulators permitted as therapeutic agents for patients with acute myeloid leukemia (AML) with resistance to conventional chemotherapy. AZA has also been approved for the treatment of chronic myelomonocytic leukemia (CMML) by the European Medicines Agency (EMA) and FDA [271].

Several studies have utilized AZA and decitabine to promote cell cycle arrest and cytotoxicity in resistant cancer cells [272,273]. In multiple myeloma, AZA reportedly leads to modulation of p16 expression, cleavage of caspase and PARP, and cell cycle arrest at G_0_/G_1_ phase [274]. Similarly, decitabine induces G_0_/G_1_ and G_2_/M arrest by modulating p21 and p38 [275]; the effectivity of decitabine at the clinical level has been well established in AML, chronic myelogenous leukemia (CML), and myelodysplasia [276].

#### Clinical trial status

A novel compound, termed SGI-110 (combination of 5-aza-2′-deoxycytidine and guanosine), functions as a prodrug for decitabine and is reported to display promising results against drug-resistant AML and myelodysplasia in Phase III clinical trials (NCT02348489) [277] (https://clinicaltrials.gov/). Both AZA and decitabine have undergone many clinical trials before being accepted by FDA for the treatment of several hematological cancers (Table 1). Currently, clinical trials are going on to elucidate their dosage and potency in treating various solid tumors. Subsequent quests for more efficacious DNMT inhibitors have led to the synthesis of MG98, a second-generation phosphorothioate antisense oligodeoxynucleotide that prevents translation of DNMT1 mRNA. MG98 is currently undergoing phase I/II clinical trials in solid tumors’ patients (NCT00003890) [278]. The clinical trial status of various DNMT inhibitors is listed in Table 1.

### HDACis as epi-drugs against chemoresistant cancers

The HDAC family constitutes a major target of the epigenome-modifying drugs as HDAC inhibition can trigger a magnitude of cellular responses, promoting intracellular stress and eventually cell death. The role of HDACs in maintaining chemoresistance has been well established by various studies [49,279,280], and the use of HDACis has been in light for several years now. The two HDACis suberoylanilide hydroxamic acid (SAHA, class I and II inhibitor) and depsipeptide (romidepsin, class I inhibitor) have been permitted by FDA for treating T-cell lymphomas [281,282]. SAHA is also reported to induce apoptosis in multiple myelomas through dephosphorylation of Rb, attenuation of Bcl2, and augmentation of p21 and p53 [283]. In multiple myeloma, a combination of Panobinostat, a non-selective pan HDACi, and bortezomib is reported to have synergistic activity against Dexamethasone resistance [284]. The active DNA repair pathway is one of the foremost causes behind chemotherapeutic resistance [285]. Studies on various HDACis in different solid and liquid cancers have shown pronounced effects against this active DNA repair system, ultimately hindering the drug resistance ability of the cancer cells. For instance, in AML, a co-treatment with HDACi belinostat and NEDD8-activating enzyme inhibitor pevonedistat reportedly inhibits expression of the DNA repair proteins 53BP1 and RAD51 [205]. In neuroblastoma, SAHA is reported to repress DNA repair-mediated acquisition of resistance by inhibiting Ku-86, a significant contributor of non-homologous end-joining DNA damage repair [286]. The pan HDACi valproic acid has been used in clinical trials singly or in combination with other drugs as a tool to target cancer cell resistance. For instance, in neuroendocrine carcinoma and central nervous system (CNS) tumors, treatment with valproic acid displayed significant stability in the patients [287,288]. Studies reporting valproic acid treatment in combination with various drugs like S-1 (a novel oral fluoropyrimidine derivative), decitabine, bevacizumab have indicated effective anti-tumor activity [289–291].

#### Clinical trial status

Compared with DNMTis, HDACis have been studied in several diseases, including hematological malignancies, solid tumors and inflammatory disorders, and have elicited positive outcomes. The three FDA-approved HDACis SAHA, romidepsin, and belinostat, are approved as a therapeutic tool in cutaneous T-cell lymphoma. They are being extensively assessed in the clinical trials for other solid and hematological cancers, either alone or co-treatment with other anti-cancer drugs. For instance, in glioblastoma patients, SAHA is currently under clinical trial combined with radiotherapy and chemotherapeutic drug temozolomide (NCT00731731). A Phase II clinical using romidepsin in peripheral T-cell lymphoma showed an overall 38% response rate [292]. According to https://clinicaltrials.gov/, approximately 30 clinical trials are ongoing with romidepsin, either as a single agent or combined with other drugs. In Phase II clinical trials, belinostat has been utilized to sensitize platinum-resistant epithelial cancer and micropapillary ovarian tumors [293]. Another phase I/II clinical trial of belinostat in thymic epithelial tumors indicated potency of belinostat-PAC (P: cis**p**latin, A: **a**driamycin/doxorubicin C: **c**yclophosphamide) co-treatment in reducing tumor resistance [294].

The preclinical and clinical studies using the different HDACis are enlisted in Table 1.

### HMT inhibitors in anti-cancer therapeutics

Reversible histone methylation plays a crucial role in the development and progression of various cancers and histone methyltransferase inhibitors (HMTis) are emerging as potential epigenome-modifying tools with promising outcomes in the arena of clinical oncology. Some of the lysine and arginine methyltransferases widely studied in anti-cancer therapeutics include G9a, EZH2, DOT1L (disruptor of telomeric silencing 1-like protein) and PRMT1 (protein arginine methyltransferases 1), inhibitors of which are being extensively investigated [295].

The expression and activity of EZH2, one of the most well-known HMTs, have been associated with the tumor progression of various cancers [295]. Enhanced EZH2 activity is reported to induce tumor growth and resistance to conventional chemotherapy, leading to poor prognostic outcomes [296]. The EZH2 inhibitor tazemetostat, the first FDA-approved HMT inhibitor for cancer treatment, is currently one of the most investigated compounds in the clinical trial, with its anti-cancer properties established in preclinical studies [297,298]. Other selective inhibitors of EZH2 such as PF-06821497, GSK2816126, are also reported to reduce tumor growth and progression in preclinical models [299,300].

EPZ-5676 or pinometostat, a specific inhibitor against H3K79 methyltransferase DOT1L, has been under preclinical investigation for some years and has shown potency in treating the leukemia model [301].

PRMTs, the HMTs of the arginine methylase family, play a crucial role in cancer signaling pathways. Different classes of PRMTs are reported to be up-regulated and play pro-cancer roles in various solid and hematological cancers [302–304]. Inhibition of PRMTs has displayed anti-cancer potential in preclinical studies. PF-06939999 and EPZ015666 (GSK3235025), potent orally bioavailable PRMT5 inhibitors, have been reported to show significant antitumor activity [305].

#### Clinical trial status

The EZH2 inhibitors tazemetostat CPI-0209, CPI-1205, GSK2816126, DS-3201, and PF-06821497 have been in clinical trials. Tazemetostat, the most investigated EZH2 inhibitor, has undergone several clinical trials in different types of cancers and has demonstrated potent anti-tumor effects (enlisted in Table 1). In patients with refractory malignant mesothelioma with BAP1 inactivation, a Phase II clinical study using tazemetostat has established its activity as an anti-cancer agent with low toxicity (NCT02860286). However, several other clinical trials of tazemetostat in various cancers are still in progress.

EPZ-5676 has been under clinical trials in patients with refractory leukemia (NCT01684150, NCT02141828) and has shown modest clinical activity and an acceptable safety profile (Table 1) [255].

Three PRMT5 inhibitors, PF-06939999, GSK3326595 and JNJ-6461978, are currently under clinical trials in patients with solid and hematologic cancers. GSK3368715, the only developed inhibitor of PRMT1, has recently entered Phase I clinical trials in patients with various solid and hematologic malignancies (NCT03666988).

### Bromodomain inhibitors as anti-cancer agents

Bromodomain (BRD) proteins are ‘readers’ of histone acetylation, which target transcription factors and chromatin-remodeling enzymes to target sites on the chromatin to regulate gene expression. BRD proteins are generally associated with the augmentation of target genes [306]. Bromo- and extra-terminal domain (BET) proteins, a crucial subfamily of BRD proteins, constitute BRDT, BRD2, BRD3 and BRD4. Due to their diverse genome-wide targets, BET proteins play significant roles in cellular survival and homeostasis, dysregulation of which leads to malignancy [307]. Over the recent years, targeting BET proteins through small molecule inhibitors has become a highlight of anti-cancer research to sensitize drug-resistant cancer cells towards therapy. JQ1 and I-BET762 (GSK525762A/molibresib/I-BET) are the two foremost BET inhibitors that have been extensively studied as cancer therapeutics over the past few years. JQ1 has been reported to induce anti-cancer effects in nuclear protein in testis (NUT) midline carcinoma (NMC), AML, medulloblastoma, breast cancer, and lung cancer [257–261]. Due to the low oral bioavailability and short half-life of JQ1, it has not been used in clinical trials [307]. OTX015 (Birabresib), another small molecule similar to JQ1, inhibits BRD2, BRD3 and BRD4 supports oral administration and has been reported to display anti-cancer effects in preclinical studies of drug-resistant neuroblastoma [264], mesothelioma [265], multiple myeloma [266], and B-cell lymphoma [267]. I-BET62 is orally bioavailable and has shown efficacy as an anti-tumor agent in preclinical models, including multiple myeloma [268], pancreatic adenocarcinoma [269], and neuroblastoma [270].

#### Clinical trial status

Consecutive research has led to the development of JQ1 analog TEN-010 (JQ2), which has completed Phase I clinical trial in various advanced solid tumors (NCT01987362), AML, and MDS patients (NCT02308761) [262,263] (Table 1). However, OTX015 ha snot produced any significant positive outcome in clinical trials as the patients administered with OTX015 displayed symptoms of dose-limited toxicities like fatigue and headache, gastrointestinal disorders, anemia, hyperbilirubinemia etc. [308,309] (Table 1). I-BET62 is currently under clinical trial to specify doses [310].

### Usage of epi-drugs in targeting CSCs

Since CSCs are the major contributors behind drug resistance within the tumor mass, several studies have utilized epi-drugs to specifically target CSCs and sensitize them towards conventional therapies to achieve significant tumor regression (Figure 2). For instance, DNMT inhibitors AZA and decitabine have been reported to selectively target CSCs of leukemia and bladder cancer, thereby improving the efficacy of chemotherapy [311,312]. Inhibition of EZH2 and BET proteins by specific inhibitors have also been reported to repress CSC-associated tumor aggressiveness in different solid cancers [313–315]. Similarly, potent HDACis including valproic acid, SAHA and trichostatin A (TSA) have been shown to selectively target CSCs and repress their self-renewal, proliferation, migratory and therapy-resistant properties [316–318]. In a nutshell, all these reports indicate towards the crucial significance of targeting CSCs to alleviate therapy resistance and achieve overall tumor regression.

## Role of epigenetic modulators in anti-tumor immunity

Immunotherapy or enhancing the patient’s immune response to eradicate cancer cells has become a breakthrough in anti-cancer therapeutics. But, a substantial percentage of nonresponding patients and systemic toxicities pose an obstacle to its therapeutic success [319]. An immunosuppressive tumor milieu can lead to epigenetic modification of tumor-associated immune cells, promoting therapeutic failure [132]. In this context, modulating epigenomic signatures of cancer cells and cancer-associated immune cells show promising prognostic outcomes in patient’s responses to immunotherapy.

Epigenetic reprogramming of tumor cells, TME, and tumor-associated immune cells play crucial roles in developing an antitumor immune response, patient’s response to immunotherapy, and overall prognostic outcome of the patient. For instance, derepression of endogenous retroviruses (ERVs) by LSD1 inhibitors have been reported to lead to accumulation of double-stranded RNAs (dsRNAs) in cancer cells, which simulates viral infection, in turn triggering antitumoral immunity through interferon response within the tumor milieu [320]. Another study has reported that dual inhibition of DNMTs and methyltransferase G9a in ovarian and hematological cancers leads to an augmentation of ERV transcripts and consequent induction of viral defense genes like IRF7 and STAT1 [321]. AZA treatment in colorectal cancer has been reported to promote the expression of interferon-response factors like OASL and IRF7 by inducing up-regulation of dsRNA and stimulation of the MDA5/MAVS/IRF7 pathway [322]. Inhibition of EZH2 in the ovarian cancer model has been reported to enhance the expression of Th1 chemokine genes (Cxcl9 and Cxcl10), promoting CD8+ T-cell infiltration and leading to the efficacy of anti-PD-L1 antibody immunotherapy [132]. Moreover, due to the crucial role of EZH2 in the differentiation, and maintenance of Treg cells and the expansion of effector T cells, makes it a potential target of immune-modulation therapy [323]. Repression of EZH2 in tumor-infiltrating Treg cells thus promote pro-inflammatory signaling and enhance recruitment of CD8+ and CD4+ effector T cells, ultimately leading to tumor regression [324].

All these reports indicate that targeting epigenetic modulators can prompt significant anti-tumor immunity and thus can serve as a complementary therapeutic approach to current conventional therapies.

## Conclusion and future perspectives

Cancer is the principal cause of global morbidity and mortality, with billions of people succumbing to it each year. The most routinely used therapeutic regimen for most advanced cancers is chemotherapy, which has led to no significant improvement in the treatment scenario and mortality worldwide. Approximately 70–80% of patients with advanced carcinomas experience recurrence, which leads to an even more aggressive form of the disease and is mostly fatal. The development of the recurrent tumor is mainly attributed to drug resistance as the therapy-resistant cells remaining after first-line therapy, serve as the seed for future relapse. Drug resistance involves a complex architecture of molecular events, including heightened drug efflux, drug inactivation, inhibition of apoptosis, and enhanced DNA repair mechanisms. This resistance towards the conventional anti-cancer stratagems, be inherent or acquired, has hindered favorable prognosis since the emergence of the chemotherapy and seems to be a prime impediment in the effective management of breast cancer. Extensive research for the past decade has established that CSCs are the primary drug-resistant cells within the tumor mass, which remains unaffected even after the chemotherapeutic cycles and finally gives rise to recurrent tumors. This indicates the importance of developing CSC targeting therapies that could sensitize the drug-resistant tumor towards conventional chemotherapy and hence will be able to complement it. Combinatorial therapies involving the usage of various drugs or pathway inhibitors and chemotherapy have been highlighted for the past few years due to their efficacy in reducing tumor burden and attenuation of drug resistance.

Epigenetic modifications play a crucial role in the acquisition of therapy resistance. However, even these combinatorial therapies often fall prey to the ingenious epigenetic reprogramming of the cancer cells, leading to treatment failure after a certain point in time. In this scenario, epigenome modifying drugs seem to be an attractive option to serve as a co-treatment procedure along with mainstream/combinatorial therapies. Hence, it is crucial to continue unravelling the intricate epigenetic regulatory mechanisms underlying the acquisition of drug resistance to keep developing more efficacious treatment possibilities. This review encompasses the currently known epigenetic landscape, which regulates the mechanisms of drug resistance in cancer. Further studies are required to enhance overall understanding and devise novel therapeutic strategies to target drug-resistant cancers.

## Abbreviations

ABC: ATP-binding cassette transporter
ALDH1: aldehyde dehydrogenase 1
AML: acute myeloid leukemia
ATM: ataxia telangiectasia mutation
AZA: 5-azacyticine
CSC: cancer stem cell
DME: drug-metabolizing enzyme
DNMT: DNA methyltransferase
DSB: double-strand break
EMT: epithelial-to-mesenchymal transition
FDA: Food and Drug Administration
GST: glutathione S-transferase
HDAC: histone deacetylase
HDACi: HDAC inhibitor
HMT: histone methyltransferase
MDSC: myeloid-derived suppressor cell
NK: natural killer
NSCC: non-stem cancer cell
ROS: reactive oxygen species
SAHA: suberoylanilide hydroxamic acid
TAM: tumor-associated macrophage
TME: tumor microenvironment
